# Supplementary material for: Assessments of effectiveness of technologies utilizations in VIHSCM among selected health facilities in Tanzania mainland
Source: PLOS Glob Public Health. 2024 Oct 17;4(10):e0002635. doi: 10.1371/journal.pgph.0002635 (PMC11486421; doi:10.1371/journal.pgph.0002635)
Supplement: Attachment — Submitted filename: Response to Reviewers_Rebattles.pdf [file pgph.0002635.s002.pdf]

To,

The Editor, Reviewers,

PLOS ONE

Date: 31st May 2024

Subject: Addressing Editor/Reviewer Comments for PGPH-D-23-02110

Dear Editor, Reviewers,

Many thanks to you for your helpful feedback. We are sorry that it took so long for us to revise. Due to the Editor's comments on the 1st draft of the Manuscript We have now tried to address all your concerns to the best of our abilities. Kindly help us with your further concerns (if any) to improve our paper.

Below we are providing our responses using the comments-response matrix and actions that we took to address them.

Best of Regards

Prof Henry A. Mollel

S/N	Comment	Reviewer's Response	Action taken	Reference	
1	Does this manuscript meet PLOS Global Public Health's publication criteria? Is the manuscript technically sound, and do the data support the conclusions? The manuscript must describe methodologically and ethically rigorous research with conclusions that are appropriately drawn based on the data presented.	Reviewer #1: Yes	No action Taken		
		Reviewer #2: Yes			
2	Has the statistical analysis been performed appropriately and rigorously?	Reviewer #1: I don't know	The statistical analysis used in the study is appropriate and was revised for clarity. We re-analysed the data using appropriate statistical methods	Refer to Table of results 1 on page 6, Table 2 on page 7, Table 3 on page 9, Table 4 on page 11 and Table 5 on page 12	
		Reviewer #2: No			

			and detailed the procedures in the revised manuscript		
3	Have the authors made all data underlying the findings in their manuscript fully available (please refer to the Data Availability Statement at the start of the manuscript PDF file)?	Reviewer #1: Yes<br>Reviewer #2: No	We have updated the Data Availability Statement to ensure all data are fully accessible. The study's dataset can be found in a public repository.	Data set will be submitted upon request from the publisher	
4	Is the manuscript presented in an intelligible fashion and written in standard English?	Reviewer #1: Yes<br>Reviewer #2: No	The manuscript has been carefully reviewed and proofread by professional language editors to ensure that, it was written in proper English and presented understandably.<br>Also, English (United Kingdom) Language specification was Employed for Language checks throughout the Article	Referring to whole Manuscript writing process	
5	Review Comments to the Author				

Reviewer #1	There are multiple stylistic and grammatical errors and inconsistencies which undermine the findings being documented. I have highlighted in yellow some of these grammatical inconsistencies in the attached file. As a first step, someone needs to go through the entire manuscript and do a thorough check of the writing style and grammar.<br><br>2. The authors have presented a textual representation of the numbers that are already presented in tables; in other words, this is a duplication of information, which readers might find unnecessary. I strongly		The entire manuscript was reviewed and edited to correct grammatical errors. Furthermore, the specific inconsistencies highlighted by the reviewers were addressed by proof reading and utilising available tools for manuscript writing like grammar.<br>Also, English (United Kingdom) Language specification was Employed for Language checks throughout the Article	Refer to the Manuscript Result sections	

	suggest that rather than repeating the numbers in a paragraph that are already in a table, the authors should focus more on analyzing the data and the implications of their findings.<br><br>The findings of this study are interesting, but the conclusions are rather bland in that the authors need to say a lot more about what these data mean for Tanzania's policy and logistics concerned with making its vaccination program highly effective. I would be happy to review a revised version of this manuscript.		Duplications in the text and tables were omitted and the tables now present the findings while the text presents the analysis and implications of the findings.<br><br>The discussions and conclusion were revised to match the findings		
Reviewer #2	This is a helpful study relevant to policymakers in Tanzania, and I'm so glad that health workers found the supply chain management system useful.<br><br>As the manuscript is written, it does not really articulate what this research is adding to the literature or knowledge on this topic that would be useful or relevant to researchers working on immunization more broadly. This could help elevate this paper to broaden its interest to those beyond Tanzania		we have revised the introduction and discussion sections to clearly articulate the study's contribution to the larger body of academic literature on supply chain management and immunization.<br><br>. We also emphasized the applicability of our findings to other low- and middle-	Refer to the Introduction section.<br><br>Refer to the discussion section as we have added the study	

			income countries and provided in-depth analysis and implications that broaden the research's interest beyond Tanzania.	implications to policy makers and all other relevant stakeholders	
Reviewer #2	The manuscript could benefit from a more targeted articulation of what information this adds to the literature and what the broad take-aways are. Some suggestions to this end:		We have revised the manuscript to include a more targeted articulation of the study's contributions to the literature	Refer to the discussion and conclusion section	
Reviewer #2	The introduction would be strengthened by clearly articulating the gap in academic knowledge that this article is filling. The practical utility of this information is clear—but what is this paper adding to the academic literature? What are the gaps in literature in supply chain management broadly, beyond Tanzania (and perhaps even beyond immunization)?		The introduction was updated to more clearly define the scientific gap that this paper fills and to emphasize how it adds to the body of knowledge on supply chain management.	Refer to the introduction part	
Reviewer #2	The manuscript would benefit from some information on the supply chain management system itself; since it seems to be useful,		Additional details about the supply chain management system were added to provide	We have added more literature reviews from papers on Vaccines Supply chain	

	readers from other countries will want that detail.		context for readers from other countries.	management from other countries.(Blasioli, Mansouri, Tamvada, & Hassini, 2023);(Blasioli et al., 2023; Canavan, Sipsma, Kassie, & Bradley, 2014; Dudeja et al., 2024)	
Reviewer #2	I was interested in some more detailed analysis that could help policymakers in Tanzania and elsewhere. Did satisfaction with the system differ, for example, depending on whether the system was electronic or on paper?		Additional analyses were incorporated to explore differences in the effectiveness of the system depending on whether it was electronic or paper-based.<br>On the matter of satisfaction of digital or paper that can be referred to Further research area.		
Reviewer #2	The tables are somewhat confusing as laid out; I was not always sure what the percentages were referring to. For example in Table 2 I initially thought that not all facilities had refrigerators; I		The tables were reformatted for clarity, with clearer labels and explanations for the percentages used	Refer to Table of results 1 on page 6, Table 2 on page 7, Table 3 on page 9, Table 4 on page 11 and Table 5 on page 12	

	think reformatting the tables could help with this.				
Reviewer #2	Qualitative interviews were mentioned but not included in the manuscript. What insights come from that material?		Qualitative interviews were synthesized in the discussion part to substantiate the analysis.		
6	Do you want your identity to be public for this peer review? If you choose “no”, your identity will remain anonymous but your review may still be made public.<br><br>For information about this choice, including consent withdrawal, please see our Privacy Policy	Reviewer #1: No<br>Reviewer #2: No			
