# Supplementary material for: Assessments of effectiveness of technologies utilizations in VIHSCM among selected health facilities in Tanzania mainland
Source: PLOS Glob Public Health. 2024 Oct 17;4(10):e0002635. doi: 10.1371/journal.pgph.0002635 (PMC11486421; doi:10.1371/journal.pgph.0002635)
Supplement: Attachment — Submitted filename: Rebattle Letter_Response to Reviewers.docx [file pgph.0002635.s003.docx]

**To,**

**The Editor, Reviewers,**

**PLOS ONE**

**Date: 21st August 2024**

**Subject: Addressing Editor/Reviewer Comments for PGPH-D-23-02110**

Dear Editor, Reviewers,

Many thanks to you for your helpful feedback. We are sorry that it took so long for us to revise.

Due to the Editor's comments on the 1^st^ draft of the Manuscript We have now tried to address all your concerns to the best of our abilities. Kindly help us with your further concerns (if any) to improve our paper.

Below we are providing our responses using the comments-response matrix and actions that we took to address them.

Best of Regards

Prof Henry A. Mollel

**Review Comments to the Author**

Please use the space provided to explain your answers to the questions above. You may also include additional comments for the author, including concerns about dual publication, research ethics, or publication ethics. (Please upload your review as an attachment if it exceeds 20,000 characters)

Reviewer #1: The abstract (content and style) on the cover page is different from the Abstract included in the manuscript. Please reconcile.

**Author Response:** *The Abstract have been updated to reflect the current content of the article*

Reviewer #2: The writing and tables in this article have been improved, but this paper still needs major work before it is ready for publication. Really articulating what this paper is adding to the existing literature will be very helpful in showing what this important work is adding.

**Author Response**: *The contribution of the paper has been articulated in the Introduction part in this paragraph “Furthermore observed that integration of VIHSCM technologies in the management of vaccine and logistical information faced multiple challenges, such as inadequate electricity, unreliable network connectivity, limited information on cold chain equipment availability, and a lack of trained and motivated health personnel. Adding to the limited literature on understanding the efficacy of current technologies in vaccinations and immunizations within healthcare supply chains constitutes a pivotal area of inquiry. This study significantly contributes to the existing body of literature by evaluating the effectiveness of technology integration in Vaccine and Immunization Health Supply Chain Management (VIHSCM) within Tanzania's healthcare system. While previous studies have acknowledged the challenges and benefits of digital transformation in healthcare supply chains, this paper provides a detailed analysis of specific technologies used in different Tanzanian health facilities, highlighting how these technologies have improved vaccine access and immunisation services to achieve targeted health outcomes.”*

***Also in the conclusion part “****Overall, the study underscores the need for continuous investment in and enhancement of digital tools to improve vaccine supply chain management and immunization outcomes in Tanzania. Addressing the specific challenges faced by different facility types will further optimize the integration of immunization programs into primary healthcare systems, thereby contributing to the achievement of universal health coverage (UHC) and the fulfilment of health-related Sustainable Development Goals (SDGs).****”***

--This paper would benefit from more information in the introduction in terms of what this study adds specifically to the literature on vaccine management systems. Then, this should be clearly articulated in the conclusion.

***Author response:*** *We have added the paragraph that explains the types of management systems that have been adopted in Tanzania and their utilizations i.e. To support this, the MoHSW's Immunization and Vaccines Development (IVD) department implemented advanced technologies, including the Vaccine Information Management System (VIMS), Warehouse Management Information Systems, and the Tanzania Immunization Registry (TImR), to enhance supply chain and logistics*

--If interview data are used in the authors' thinking, that is wonderful. If so, that interview data should be presented in the results, not in discussion. They should be integrated into the discussion with quotes, for example in 3.4. If they are not used in the paper, that should be noted in the methods.

***Author Response:*** *Interview data are not used in this study we only used the questionnaire data*

--The paper needs major attention to formatting and proofreading. For example, an abstract seems to have been inserted into the methods on p. 6. A careful proofreading of the entire document is needed. There are many grammatical errors and some references are not formatted correctly.

***Author Response:*** *We have worked on the grammatical errors using tools like Grammarly and Paper Pal also the article is the product of several authors who are competent and well-equipped.*

--p. 4, interesting information on the sharp decrease in immunization coverage in 2022. Can this be drawn out a bit more, as the issues listed did not seem to explain it (wouldn’t these have been issues before)? Is this about COVID, or some political dynamics? How is this related (or not related) to the topic of this paper?

***Author Response:*** *we have addressed the comment as viewed by the reviewer that the disruption in Immunization services coverage was due to COVID-19 The decline in fully vaccinated children since 2015–16 is mainly due to limited capacity, inaccurate target populations, poor vaccine supply visibility, and challenges in tracking immunisation defaulters worsened by the COVID-19 outbreak, (Dolan, 2020; Dudeja et al., 2024; TDHS, 2023; WHO, 2014).*

--The formatting of Table 2 still appears incorrect. The percentages seem to be wrong across the columns. How can less than half of all facilities have refrigerators, but then it somehow adds up to 100%?

***Author Response:*** *The table has been rectified by reducing the variable dimensions2 and other tables dimensions in terms of describing the analysis to be precise in terms of explanations.*

--Throughout the results, rather than repeating what is in the tables, I suggest using the narrative to hit on and discuss the key points, perhaps with additional information from the interviews. Much of the discussion could be shortened (eg., it’s fine to just state that refrigerators are everywhere and then move on).

***Author Response:*** *we have worked on the Review comments by shortening the descriptions of the results*

--The formatting of Table 4 is still very confusing; can this be reformatted for clarity?

***Author Response:*** *The table 4 has been rectified by breaking the table into three other tables that will improve clarity in describing the analysis to be precise in terms of explanations of the results of the study.*

7. PLOS authors have the option to publish the peer review history of their article ([what does this mean?](https://journals.plos.org/globalpublichealth/s/editorial-and-peer-review-process#loc-peer-review-history)). If published, this will include your full peer review and any attached files.


**Do you want your identity to be public for this peer review?** If you choose “no”, your identity will remain anonymous but your review may still be made public.


For information about this choice, including consent withdrawal, please see our [Privacy Policy](https://www.plos.org/privacy-policy).

Reviewer #1: No

Reviewer #2: No

[NOTE: If reviewer comments were submitted as an attachment file, they will be attached to this email and accessible via the submission site. Please log into your account, locate the manuscript record, and check for the action link "View Attachments". If this link does not appear, there are no attachment files.]

While revising your submission, please upload your figure files to the Preflight Analysis and Conversion Engine (PACE) digital diagnostic tool, <https://pacev2.apexcovantage.com/>. PACE helps ensure that figures meet PLOS requirements. To use PACE, you must first register as a user. Registration is free. Then, login and navigate to the UPLOAD tab, where you will find detailed instructions on how to use the tool. If you encounter any issues or have any questions when using PACE, please email PLOS at [figures@plos.org](mailto:figures@plos.org). Please note that Supporting Information files do not need this step.

**Author Response**: *We have uploaded the table of the manuscript in the PACE platform*
